# A Rare Case of Primary Cutaneous Adenoid Cystic Carcinoma

**DOI:** 10.3390/diagnostics15050533

**Published:** 2025-02-21

**Authors:** Ioannis Katsarelas, Afroditi Kotarela, Mattheos Bobos, Alexandra Panagiotou, Periklis Dimasis

**Affiliations:** 1 Department of General Surgery, General Hospital of Katerini, 60100 Katerini, Greece; afrodite.kot@gmail.com (A.K.); alexandrapanayiotou.larissa98@gmail.com (A.P.); dimasis@yahoo.com (P.D.); 2 Department of Biomedical Sciences, School of Health Sciences, International Hellenic University, Alexandrian Campus, 57400 Thessaloniki, Greece; mbobos@icloud.com

**Keywords:** skin tumors, primary cutaneous adenoid cystic carcinoma, dermatologic surgery, pathology

## Abstract

Primary cutaneous adenoid cystic carcinoma (PCACC) isa rare skin malignancy first reported in the 1970s with limited number of cases found in the literature. These neoplasms are typically identified in middle-to-older-age individuals and are mostly located in the scalp and neck region but can identified throughout the body. We describe the case of a 67-year-old male patient that presented to our department with a slow-growing nodule in the left gluteal region that turned out to be a PCACC and analyze the differential diagnosis, radiology, histopathological findings and successful treatment with a wide local excision. Current literature on the subject is also presented and discussed.

**Figure 1:**
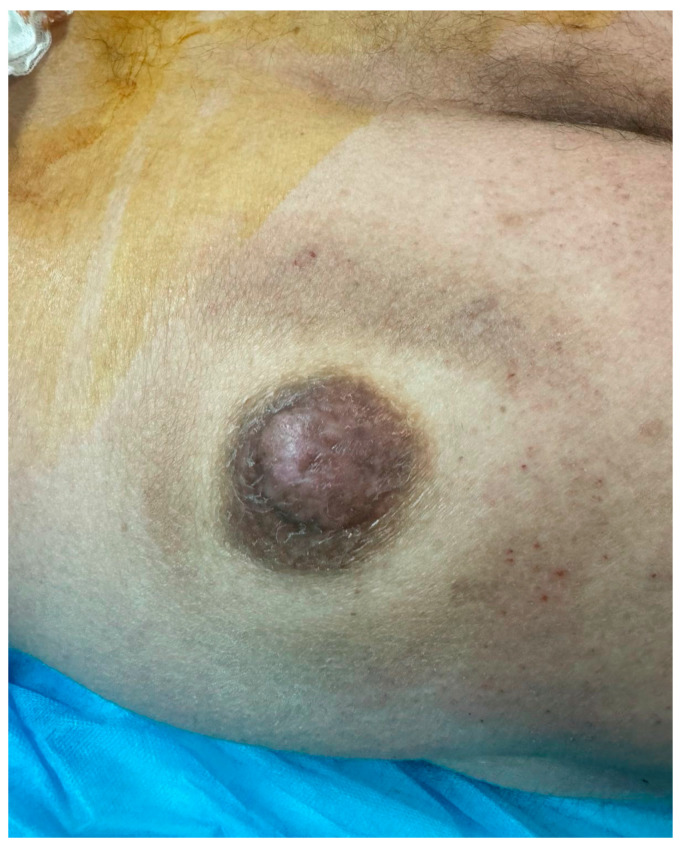
Adenoid cystic carcinomas are malignancies thought to arise from secretory glands throughout the body, mostly from minor or major salivary glands [1]. Primary cutaneous adenoid cystic carcinoma is a rare tumor, with less than 500 cases reported worldwide [2], originating from the sweat glands of the skin and predominantly located in the scalp, face and neck region [3]. They represent around 1% of skin tumors located in these areas. They can also be found in other skin sites such as the chest and abdomen. Patients typically present with large (around 4 cm), skin-colored, slow-growing and painless lesions [4]. A 67-year-old male presented to the Outpatient Surgical Clinic of General Hospital of Katerini, complaining about a large skin nodule located in his left glute that he had noticed growing over the last several months. He did not mention any other symptoms. Upon physical examination, a projecting, round-shaped lesion was revealed which was firm on palpation, had a gray-tan color compared to the darker ring-like surrounding skin and was 3.5 cm in length. The rest of his cutaneous exam was normal. No lymphadenopathy was presented. No personal or family history was reported. Our initial differential diagnosis was adnexal tumors and nodular basal cell carcinoma.

**Figure 2:**
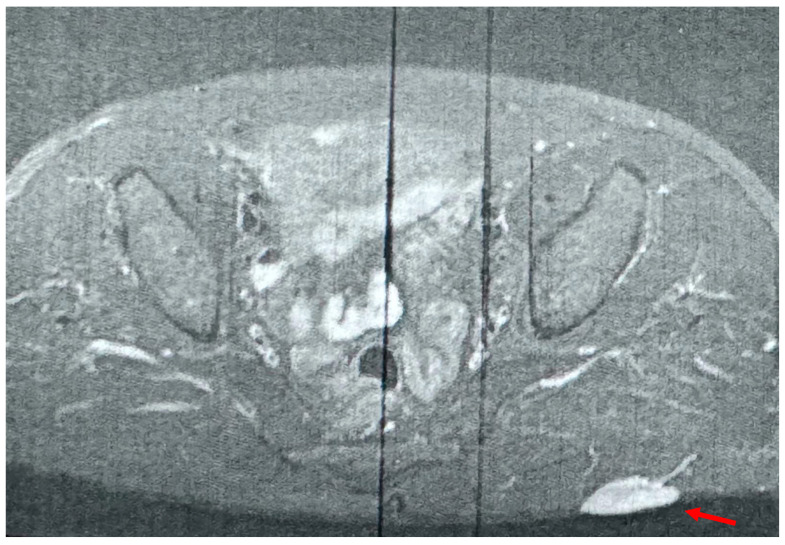
Due to the lesion being highly suspicious of malignancy, an MRI was proposed to identify its local growth and potential characteristics. The radiology report from the MRI characterized the lesion (red arrow) as benign, suggesting that it could be a hemangioma.

**Figure 3:**
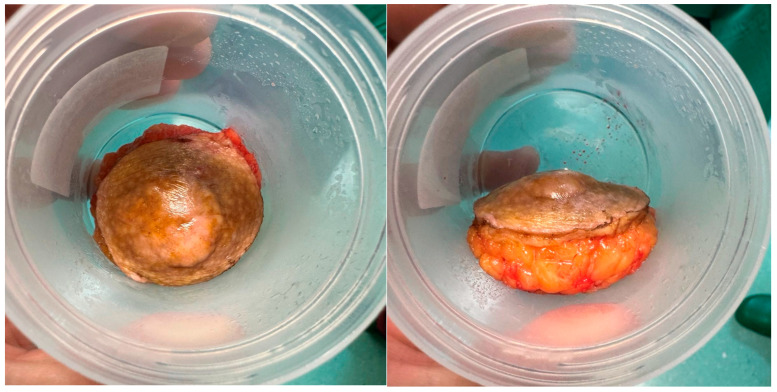
The patient was then scheduled for a local excision of the lesion. We considered 5 mm margins because of the tumor size and the benign radiology characteristics. It was performed in the operating theater under local anesthesia. The surgical specimen was skin of 5.5 cm length, round-shaped and with a width of 2 cm including subcutaneous fat, which the lesion was invading.

**Figure 4:**
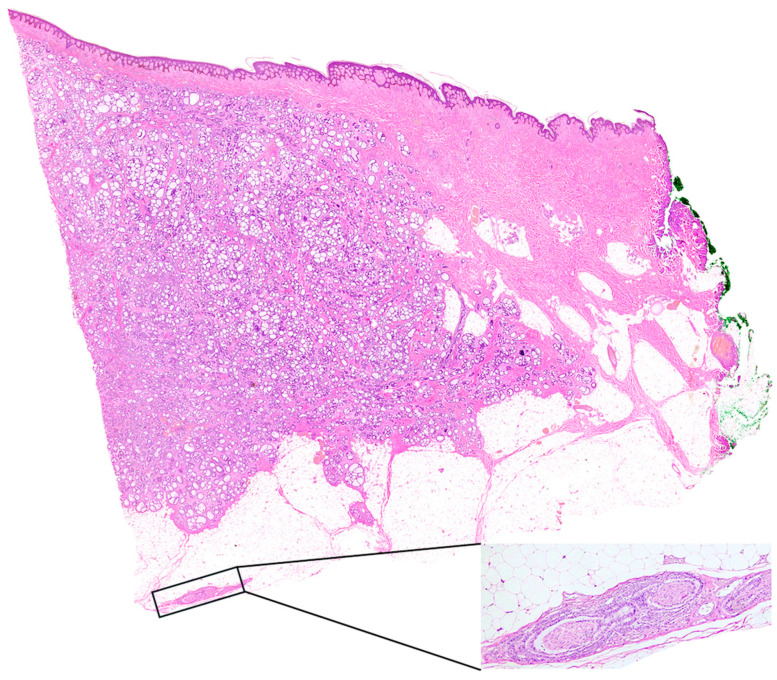
Whole slide image shows invasion of the dermis and subcutaneous adipose tissue from monomorphic basaloid cells arranged in tubules and nests, mainly with a cribriform growth pattern. Perineural and intraneural invasion is also noted in subcutaneous adipose tissue (Stain: Hematoxylin and Eosin). Histopathology classified the lesion as adnexal carcinoma of the skin and specifically as cutaneous adenoid cystic carcinoma which did not invade the epidermis. Other findings included no vascular invasion and mild inflammation. Invasion depth was 18 mm and was classified as very high risk (NCCN guidelines version 1.2024) and stage V (Clark classification). Resection margins were 5 mm (side) and 9 mm (depth). Immunohistochemistry or genetic testing was not performed at that time. Following the histopathology report, it was necessary to distinguish if the neoplasm was primary or metastasis from another site. The patient was subjected to otorhinolaryngological examination and CT scans of the head, neck, thorax and abdomen, with no findings of another primary site, which confirmed the diagnosis of PCACC. Serum carcinoembryonic antigen was also measured and found to be within normal range. Treatment was concluded with a second operation which consisted of wide surgical excision with at least 2 cm margins. The histopathology report came back with no evidence of residual tumor. In follow-up visits after 1.5 years, the patient remains recurrence-free with no evidence of metastasis. Primary cutaneous adenoid cystic carcinoma is a rare skin malignancy first described in 1975 by Boggio [5]. This tumor is primarily identified in individuals over the age of 60 years old, slightly more common in women [2]. PCACC typically presents as slow-growing, rarely ulcerated, painless, solid to cystic, skin-colored tumors, 0.5–8.0 cm in diameter on the head, face, breast, external auditory canal, upper thorax, major bronchi, uterine cervix, upper extremities and skin. The scalp, neck and thorax have been the most common sites of presentation. In order to confirm the diagnosis of PCACC, it is essential to rule out the possibility of the lesion being metastatic from another primary site because adenoid cystic carcinomas (ACC) can arise from a variety of primary sites such as the salivary glands, respiratory tract, cervix, vulva, breast, thymus, prostate, external auditory canal, esophagus and skin, with the major and minor salivary glands being the main sites of appearance. ACC’s can metastasize to the regional lymph nodes, lungs, bone and brain [3]. Although PCACC’s are less aggressive compared to ACC’s, metastasis in cases of the former have been reported. PCACC is locally invasive cancer with 70% being confined to the skin; 25% had regional metastasis and 5% distant metastasis [6]. Naylor et al. report a 44% recurrence rate in a 5-year follow up, and the 10-year survival rate is as high as 96% [7]. Risk factors are unclear as of now, but studies suggest that somatic gene mutations are linked to the neoplasm and especially gene MYB-NFIB, suggestive of the correlation between PCACC’s and salivary gland carcinomas [8]. Clinical or radiological findings alone are not specific enough for the tumor and only histopathology can confirm the diagnosis. Dermoscopy of PCACC can be presented as a well-circumscribed homogeneous erythematous nodule, arborizing vessels with erosions and hemorrhagic suffusions. Other rare dermoscopic findings of adenoid cystic lesions could include follicular openings, whitish veil and pepper-like gray dots, all the above being rare in PCACC and not distinctive. Shared characteristics with other adenoid skin tumors mean that dermoscopy has little value in the diagnosis, the gold standard of which is histology. Its typical histological findings consist of dermal islands of basaloid cells arranged in a cribriform pattern with ‘punched-out’ pseudocysts filled with mucin (Swiss cheese pattern). Tumor cells are basaloid, with several mitotic figures, no true palisading and a tendency to show perineural infiltrates, particularly at the periphery, which is characteristic for this tumor [9]. Perineural invasion is very common in PCACC and has been associated with a higher probability of local recurrence [7]. Therefore, adequate distinction of PCACC from other skin tumors that could be part of the differential diagnosis, such as basal cell carcinoma with adenoid cystic differentiation, cylindroma, mucinous apocrine carcinoma and apocrine mixed tumor of skin and metastasis arising from other malignancies with histological features of ACCs, is essential for the proper management of the disease. Wide surgical excision with at least 2 cm tumor-free margins is the treatment of choice to lessen the possibility of a local recurrence, and a long-term follow-up is essential because of the percentage of recurrences reported. Lymph node excision is not advised if not for lymphadenopathy, while adjuvant radiotherapy and chemotherapy are not the standard of care and should be considered only in metastatic disease. Mohs surgery should also be considered and has exhibited good results [10]. In conclusion, PCACC is a rare skin malignancy that should be considered in adnexal neoplasm differential diagnosis. Careful physical examination, histopathology and imaging are used to confirm such diagnosis. Poorer outcomes were reported with PCACC being diagnosed in an advanced stage or age. Therefore, doctors need to be alert of this malignancy and proceed to proper work-up and treatment. Our case displays many of the characteristics found in the literature, proceedings that the patient should undergo and highlights the need for either dermatologists or surgeons to be cautious even in cases of not thunderous symptoms or benign radiology findings in order to treat the malignancy as soon as possible and to prevent recurrence or metastatic disease. Future research should be conducted to form a better understanding of the neoplasm and its characteristics because of the limited number of cases reported in the past 50 years.

## Data Availability

All relevant data are within the manuscript.
